# Outcome and Clinical Characteristics of Nosocomial Infection in Adult Patients Undergoing Extracorporeal Membrane Oxygenation: A Systematic Review and Meta-Analysis

**DOI:** 10.3389/fpubh.2022.857873

**Published:** 2022-06-24

**Authors:** Xiyuan Li, Liangshan Wang, Hong Wang, Xiaotong Hou

**Affiliations:** ^1^Center for Cardiac Intensive Care, Beijing Institute of Heart, Lung and Blood Vessel Diseases, Beijing Anzhen Hospital, Capital Medical University, Beijing, China; ^2^Department of Intensive Care Unit, Aviation General Hospital of China Medical University, Beijing, China

**Keywords:** extracorporeal membrane oxygenation, cross infection, epidemiology, risk factors, system review, meta-analysis

## Abstract

**Objective:**

This study conducts a meta-analysis of clinical outcomes of nosocomial infection in adult patients receiving extracorporeal membrane oxygenation (ECMO) and systematically evaluates clinical epidemiological characteristics.

**Methods:**

Document retrieval strategies were determined, and all adult patients treated by ECMO were included. The prevalence, incidence, mortality, ECMO use time, intensive care unit (ICU) stay time, hospital stay time, and risk factors of nosocomial infection were systematically evaluated. Subsequently, a meta-analysis of the impact of nosocomial infection on risk of in-hospital mortality was conducted.

**Results:**

A total of 25 retrospective studies were included, and 19 studies were included in the meta-analysis. The prevalence of nosocomial infection was 8.8–64.0%, incidence was 1.7–85.4‰ (per 1,000 ECMO days), and in-hospital mortality was 31.5–75.4%. The duration of ECMO usage and length of ICU stay were longer for infected patients. Compared with non-infected patients, the meta-analysis revealed that nosocomial infection increased the relative risk of death of adult patients receiving ECMO by 32%. The risk factors included the duration of ECMO usage and disease severity score.

**Conclusions:**

Adult patients treated by ECMO have high prevalence of nosocomial infection. In addition, their ECMO use time and ICU stays are longer. Nosocomial infection significantly increases the relative risk of in-hospital mortality.

## Introduction

Since the first successful clinical application of extracorporeal membrane oxygenation (ECMO) technology in 1972 (1), the application of ECMO in adult severe cardiopulmonary failure has gradually increased. For more than 20 years, ECMO has played a vital role in the rescue of refractory cardiogenic shock, severe respiratory failure, and cardiac arrest.

Although clinicians have accumulated experience in the application of ECMO, many patients treated by ECMO are accompanied by life-threatening complications (bleeding, thrombosis, and infection), and the mortality is still very high. Nosocomial infection is a common complication in patients treated by ECMO (2). To date, there is no unified understanding of ECMO-related nosocomial infection from diagnosis to treatment to prevention.

The epidemiological study on ECMO-related nosocomial infection does not have sufficient data support from multicenter and prospective studies (2). Based on data of the Extracorporeal Life Support Organization (ELSO) (3, 4), retrospective studies revealed that the overall prevalence of nosocomial infection in adult patients treated by ECMO was 20.9%, incidence of infection was 30.6/1,000 ECMO days, and the mortality of infected patients was significantly higher than that of non-infected patients (57.6 vs. 41.5%, *P* < 0.001). However, these two retrospective studies came from the same data of ELSO. Many adult cases with incomplete data were excluded in the two studies, so the results were inevitably controversial.

The prevalence, incidence, mortality, and risk factors of nosocomial infection in patients treated by ECMO in every single center were reported with considerable discrepancy, especially in in-hospital mortality. Eight studies (3–10) concluded that nosocomial infection significantly increased in-hospital mortality; but the other studies (11–24) argued that there was no significant difference in in-hospital mortality between infected groups and uninfected groups.

This study's purpose is to conduct a meta-analysis to clarify whether nosocomial infection increased in-hospital mortality in adult patients receiving ECMO treatment and to summarize clinical epidemiological characteristics *via* a narrative systematic review.

## Information and Methods

### Study Retrieval Strategy

The PubMed, Embase, Cochrane Library, and Web of Science databases were searched online with computer to query the target studies. Retrieval time range is from January 1, 1972, to March 15, 2021. Retrieval words included the following: “extracorporeal membrane oxygenation/exp,” “extracorporeal life support,” “cross infection/exp,” “nosocomial infection,” “ventilator-associated pneumonia,” “bloodstream infection,” “sepsis,” “urinary tract infection,” “device-associated infection,” and “hospital-acquired infection.” Study screening was conducted manually.

### Definition, Inclusion, and Exclusion Criteria

#### Definition

According to the definition of nosocomial infection issued from American Center for Disease Control and Prevention (25), the nosocomial infection was defined as the infection that patients acquired in hospital according to clinical manifestation and laboratory examination, including the new infection occurred after admission, or within 30 days during receiving medical care, or within 90 days after surgery.

#### Inclusion Criteria

(1) Study population: patients over 16 years old, male or female, receiving ECMO treatment. (2) Study type: published retrospective or prospective study. (3) Interventions: no additional interventions. (4) Outcome indicators: primary outcome indicators were the incidence, prevalence, and in-hospital mortality of nosocomial infection, and secondary outcome indicators were etiological characteristics, risk factors, and related clinical characteristics of nosocomial infection.

#### Exclusion Criteria

(1) Patients who had been co-infected before receiving ECMO treatment. (2) Patients treated with combined ventricular assist devices. (3) Studies on animal experiments or studies whose full text could not be obtained. (4) Studies published in non-English languages.

### Data Extraction

Two researchers (Xiyuan Li and Liangshan Wang) independently included and cross-checked the studies according to the inclusion criteria. In case of a disagreement, a third author was consulted to assist in determination. The following information was collected from each study: first author, publication year, country, sample size, research year, definition of nosocomial infection, ECMO type, first cause of etiology, duration of ECMO usage, average length of hospital stay, prevalence, incidence, in-hospital mortality, and any independent risk factor. Prevalence of nosocomial infection = number of infected patients observed/ total number of patients observed in the same period × 100%. Incidence of nosocomial infection = number of infected patients observed/per 1,000 ECMO use days × 1000‰.

### Quality Evaluation of Studies

The Newcastle–Ottawa Scale (NOS) (2) was used for comprehensive bias risk assessment, and eight items of the following three aspects were scored: study population selection, comparability, and outcome measurement. The full score of the NOS is 9, a score of ≥7 points indicates high-quality study, a score of 5–6 points indicates medium-quality study, and a score of ≤ 4 points indicates low-quality study. A funnel plot was adopted to evaluate publication bias (Table 1).

**Table 1:** Newcastle–Ottawa Scale (NOS) evaluation of the included studies.

**Research_years**	**Study population selection**				**Comparability**		**Outcome**			**Total score**
	**Exposure** **group** **selection**	**Non** **exposure** **group** **selection**	**Method for** **determining** **exposure**	**There are no** **outcome** **events at the** **beginning of** **the study**	**Controlling** **major** **confounding** **factors**	**Controlling** **other** **confounding** **factors**	**Adequate** **evaluation**	**Adequate** **follow-up**	**Follow up** **integrity**	
Burket_1999 (11)	1	1	1	1	0	1	1	0	0	6
Hsu_2009 (12)	1	1	1	1	0	1	1	0	0	6
Sun_2010 (13)	1	1	1	0	0	1	1	0	0	5
Bizzarro_2011 (3)	0	1	0	0	0	0	1	0	0	2
Vogel_2011 (4)	0	1	0	0	0	0	1	0	0	2
Schmidt_2012 (14)	1	1	1	1	1	1	1	0	0	7
Pieri_2013 (15)	1	1	0	0	0	1	1	0	0	4
Aubron_2013 (16)	1	1	1	0	0	1	1	0	0	5
Kim_2015 (5)	1	1	1	1	0	1	1	0	0	6
Haneke_2016 (26)	1	1	0	0	0	1	1	0	0	4
Grasselli_2017 (6)	1	1	1	1	0	1	1	0	0	6
Kim_2017 (17)	1	1	1	1	0	1	1	0	0	6
Kutlesa_2017 (7)	1	1	1	0	1	1	1	0	0	6
Juthani_2018 (18)	1	1	1	0	0	1	1	0	0	5
Li_2018 (27)	1	1	1	0	0	1	0	0	0	4
Allou_2018 (19)	1	1	1	1	0	1	1	0	0	6
Silvetti_2018 (20)	1	1	1	1	1	1	1	0	0	7
Bougle_2018 (8)	1	1	1	1	1	1	0	0	0	7
Menaker_2019 (21)	1	1	1	1	0	1	1	0	0	6
Wang JR_2020 (22)	1	1	1	0	0	1	1	0	0	5
Yun_2020 (28)	1	1	1	0	0	1	0	0	0	4
Ko_2020 (23)	1	1	1	1	1	1	1	0	0	7
Selcuk_2020 (24)	1	1	1	1	0	1	1	0	0	6
Wang JR_2020 (9)	1	1	1	0	0	1	1	0	0	5
Wang_2021 (10)	1	1	1	1	1	1	1	0	0	7

### Statistical Analysis

A total of 25 studies were included, all of which were retrospective and observational. In addition, the overall quality was low. There was obvious heterogeneity in time, region, etiological composition, disease severity, etc. Therefore, the statistical analysis performed in this study was primarily descriptive statistics. For studies with NOS scores ≥ 5, a meta-analysis was conducted on in-hospital mortality. Measurement data were expressed as mean ± standard deviation (SD) when they were normally distributed; those not normally distributed were expressed as median ± quartile. Count data were expressed as n (%). Incidence of nosocomial infection was expressed as number of cases per 1,000 days. The meta-analysis was conducted using the RevMan5.3 software. Risk ratio (RR) was used as the effective index, and 95% confidence interval (CI) was reported. The Q test was conducted to analyze heterogeneity among the studies (test level was 0.1), and heterogeneity was determined with *I*^2^. If there was heterogeneity, a sensitivity analysis was conducted to check the source of heterogeneity; therefore, studies with high heterogeneity were eliminated, and effect size was remerged. If heterogeneity was acceptable (*P* ≥ 0.1, *I*^2^ < 50%), combined RR effect value was calculated using the fixed-effects model. Stata15.1 was used to draw the Starchart and Egger test, and the final studies included in the meta-analysis were tested for publication bias. *P* ≤ 0.05 was considered statistically significant.

## Results

### Study Retrieval Results

**Figure 1:**
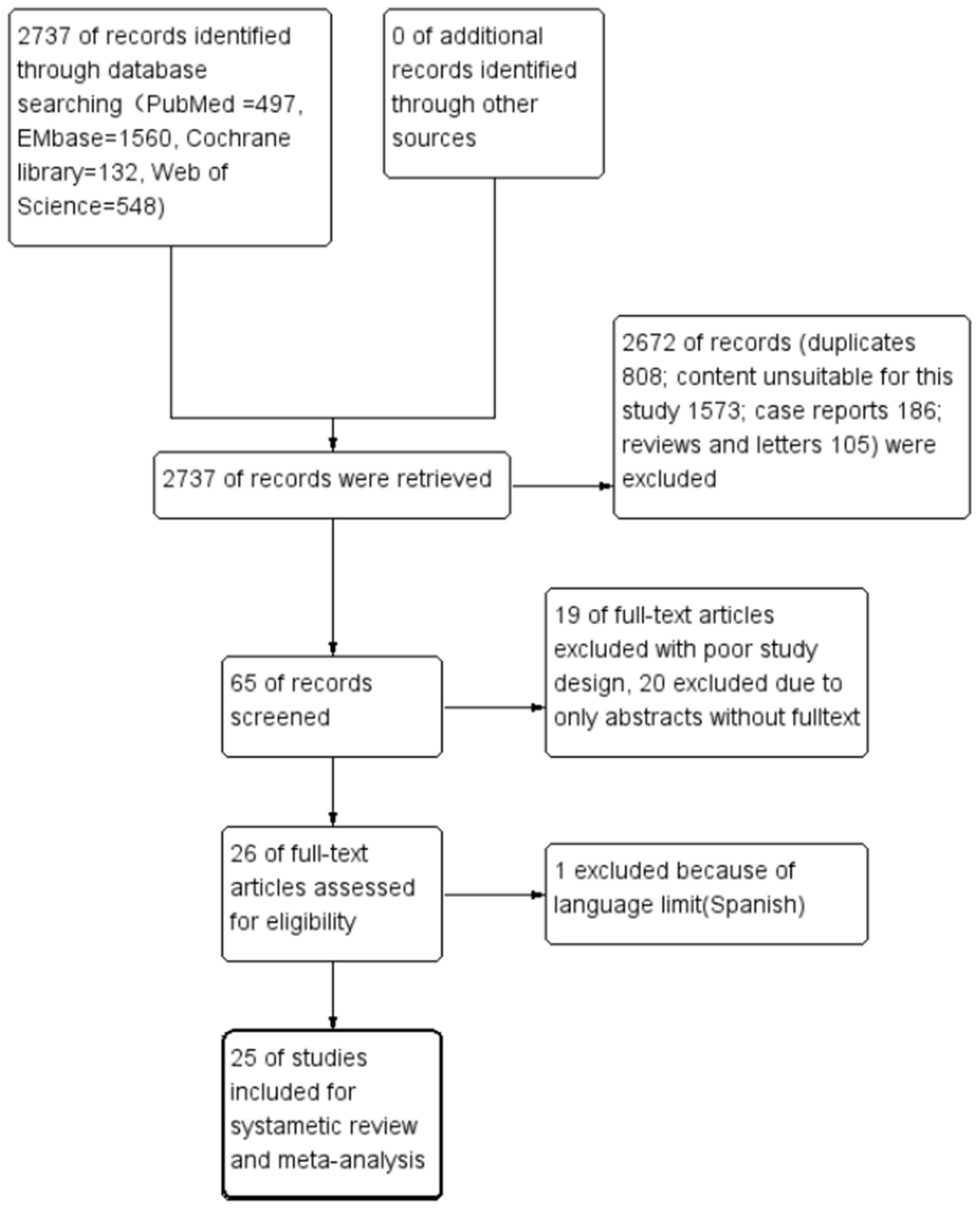
Flowchart of study screening.

A total of 2,737 related studies were retrieved in the primary screening. According to the inclusion and exclusion criteria, a total 25 studies were included, all of which were retrospective and observational. The flow chart of study screening is presented in Figure 1. The 25 studies involved a total of 9,621 cases. Among these 25 studies, two were retrospective multicenter research data released by the ELSO, reusing the same set of data (3, 4). The remaining 23 were studies from single-center institutes (Table 2).

**Table 2:** List of basic information of the studies included.

**Author**	**Year of** **publication**	**Country**	**Research** **type**	**Sample size**	**Research** **year**	**Infection group**		**Non infection group**		**Difference in** **in-hospital mortality** **between the two** **groups**
						**Survival**	**Death**	**Survival**	**Death**	
Burket et al. (11)	1999	USA	Retrospective	71	1985-1995	13	19	103	88	No
Hsu et al. (12)	2009	China	Retrospective	114	2001-2007	1	9	27	77	No
Sun et al. (13)	2010	China	Retrospective	334	1996-2007	11	34	95	194	No
Bizzarro et al. (3)	2011	USA	Retrospective	2298	1998-2008	NR	NR	NR	NR	Yes
Vogel et al. (4)	2011	USA	Retrospective	2996	1998-2008	NR	NR	NR	NR	Yes
Schmidt et al. (14)	2012	France	Retrospective	220	1987-2009	72	70	48	30	No
Pieri et al. (15)	2013	Italy	Retrospective	46	2003-2009	9	19	18	15	No
Aubron et al. (16)	2013	Australia	Retrospective	146	2009-2011	12	9	85	40	No
Kim et al. (5)	2015	Korea	Retrospective	47	2005-2011	1	12	28	6	Yes
Haneke et al. (26)	2016	Germany	Retrospective	88	2008-2014	NR	NR	NR	NR	NR
Grasselli et al. (6)	2017	Italy	Retrospective	92	2010-2015	31	21	21	8	Yes
Kim et al. (17)	2017	Korea	Retrospective	61	2011-2015	2	12	17	30	No
Kutlesa et al. (7)	2017	Croatia	Retrospective	100	2009-2016	13	22	43	22	Yes
Juthani et al. (18)	2018	USA	Retrospective	100	2012-2015	13	13	45	29	No
Li et al. (27)	2018	China	Retrospective	74	2012-2015	NR	NR	NR	NR	NR
Allou et al. (19)	2018	France	Retrospective	220	2010-2016	20	19	96	85	No
Silvetti et al. (20)	2018	Italy	Retrospective	92	2013-2017	4	10	22	56	No
Bougle et al. (8)	2018	France	Retrospective	152	2013-2014	46	39	54	13	Yes
Menaker et al. (21)	2019	USA	Retrospective	268	2010-2015	14	12	159	83	No
Wang et al. (22)	2020	China	Retrospective	69	2013-2019	8	11	32	18	No
Yun et al. (28)	2020	Korea	Retrospective	1100	2009-2016	NR	NR	NR	NR	NR
Ko et al. (23)	2020	Korea	Retrospective	150	2010-2018	19	16	62	53	No
Selcuk et al. (24)	2020	Turkey	Retrospective	126	2012-2016	9	18	16	17	No
Wang JR et al. (9)	2020	China	Retrospective	69	2013-2019	4	10	40	15	Yes
Wang et al. (10)	2021	China	Retrospective	322	2012-2017	54	77	103	88	Yes

*NR, no report*.

### Studies' Quality Evaluation Results

Most of the studies included were of medium quality; six of them had a score of less than 5 points, and the highest NOS score was only 7 points. Follow-up items were not scored in the outcome evaluation of all the studies.

### Prevalence, Incidence, and Mortality of Nosocomial Infection in Patients Receiving ECMO

Overall, majority of the 25 studies included patients treated with all types of ECMO (data extracted from documents were provided as Supplementary Materials). However, six studies included only venoarterial ECMO (VA ECMO) patients, and one study included only veno-veous ECMO (VV ECMO) patients. A total of 11 studies focused on all reports related to nosocomial infection. In adult patients receiving ECMO, the prevalence rate of nosocomial infection was 8.8–64.0%, incidence was 1.7–85.4‰ (per 1,000 ECMO days), and in-hospital mortality was 31.5–75.4%. Five studies focused on reports related to bloodstream infection. In patients treated by ECMO, the prevalence of bloodstream infection was 5.7–35.0%, incidence was 8.0–33.6‰, and in-hospital mortality was 42.0–71.7% (Table 3). In addition, two studies reported on pneumonia alone, and one study reported the corresponding proportion of catheter-related bloodstream infections.

**Table 3:** Review table of prevalence, incidence, and mortality of nosocomial infection in patients receiving extracorporeal membrane oxygenation (ECMO).

**Author**	**Publish date**	**Main study outcomes**	**Ratio of V-A mode**	**Prevalence**	**Incidence**	**In-hospital mortality**
Burket et al. (11)	1999	All	46.8	45.0	40.5	49.3
Hsu et al. (12)	2009	All	NR	8.8	NR	75.4
Sun et al. (13)	2010	All	NR	13.5	21.49	68.3
Schmidt et al. (14)	2012	All	100	64.0	75.5	45.5
Aubron et al. (16)	2013	All	66.0	24.7	30.1	34.2
Grasselli et al. (6)	2017	All	63.0	55.0	50.4	31.5
Kim et al. (17)	2017	All	34.4	23.0	43.3	68.9
Juthani et al. (18)	2018	All	36.0	26.0	NR	42.0
Ko et al. (23)	2020	All	100	23.33	1.7	46.0
Selcuk et al. (24)	2020	All	96.8	45.0	57.0	58.3
Wang et al. (10)	2021	All	100	40.68	85.4	51.2
Bougle et al. (8)	2018	Pneumonia	100	55.90	60.6	45.4
Wang et al. (9)	2020	Pneumonia	84.1	20.30	24.7	36.2
Allou et al. (19)	2018	Catheter-associated infection	68.6	17.7	17.2	47.3
Kim et al. (5)	2015	Bloodstream infection	46.8	27.7	NR	61.7
Kutlesa et al. (7)	2017	Bloodstream infection	0	35.0	NR	44.0
Silvetti et al. (20)	2018	Bloodstream infection	100	15.2	24.7	71.7
Menaker et al. (21)	2019	Bloodstream infection	45.9	VV:13.1	8.0	47.4
				VA:5.7	8.0	-
Wang et al. (22)	2020	Bloodstream infection	84.1	27.5	33.6	42.0

*NR, No report*.

A total of 12 studies reported the prevalence of both lower respiratory tract infection and bloodstream infection. Among them, six studies considered lower respiratory tract infection to be the most common (8, 10, 16, 18, 23, 24); the prevalence ranged from 12.7–55.9%. Four studies considered bloodstream infection to account for the largest proportion of complications (11, 13, 14, 22); the prevalence was 11.38–27.59%.

### Etiological Distribution of Nosocomial Infection in Patients Receiving ECMO

**Figure 2:**
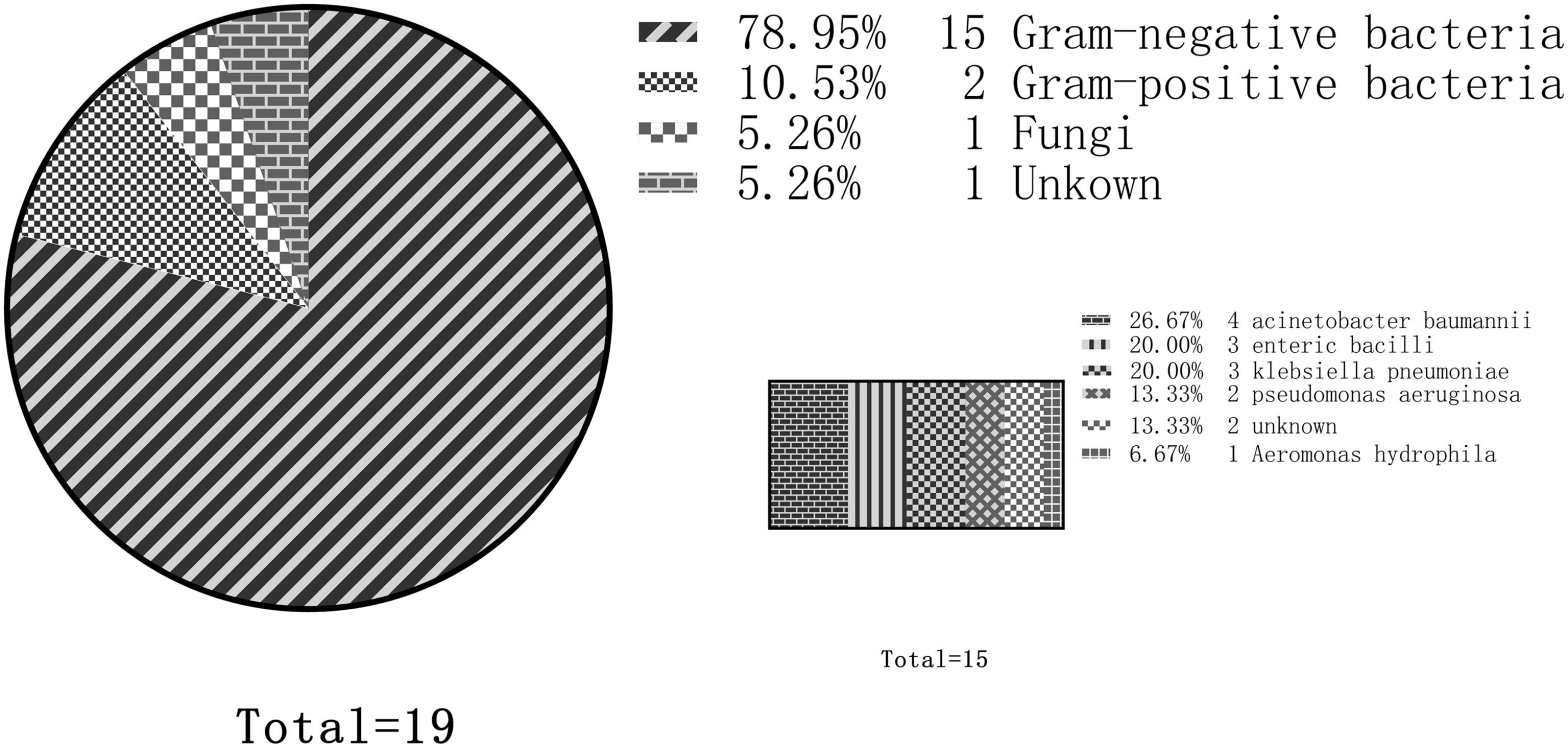
Distribution of nosocomial infection etiology in adult patients treated by extracorporeal membrane oxygenation (ECMO). Out of the 19 total studies, 15 studies reported that gram-negative bacteria were the predominant pathogens. Among them, the most common were *Acinetobacter baumannii* (four studies), followed by Enterobacter (three studies), Klebsiella pneumoniae (three studies), and Pseudomonas aeruginosa (two studies).

A total of 19 studies reported etiological results, and 15 of them reported that main pathogens isolated were Gram-negative bacteria. The most common bacteria were *Acinetobacter baumannii* (four studies), *Enterobacter* (three studies), *Klebsiella pneumoniae* (three studies), and *Pseudomonas aeruginosa* (two studies). Two studies reported that the etiology was primarily positive cocci, one study reported that the etiology was primarily *Staphylococcus*, and the other study reported that the etiology was primarily *Group B streptococcus*. In addition, two studies reported that nosocomial infection was primarily a fungal infection, mainly *Candida* (Figure 2).

### General Clinical Characteristics of Patients Receiving ECMO

**Figure 3:**
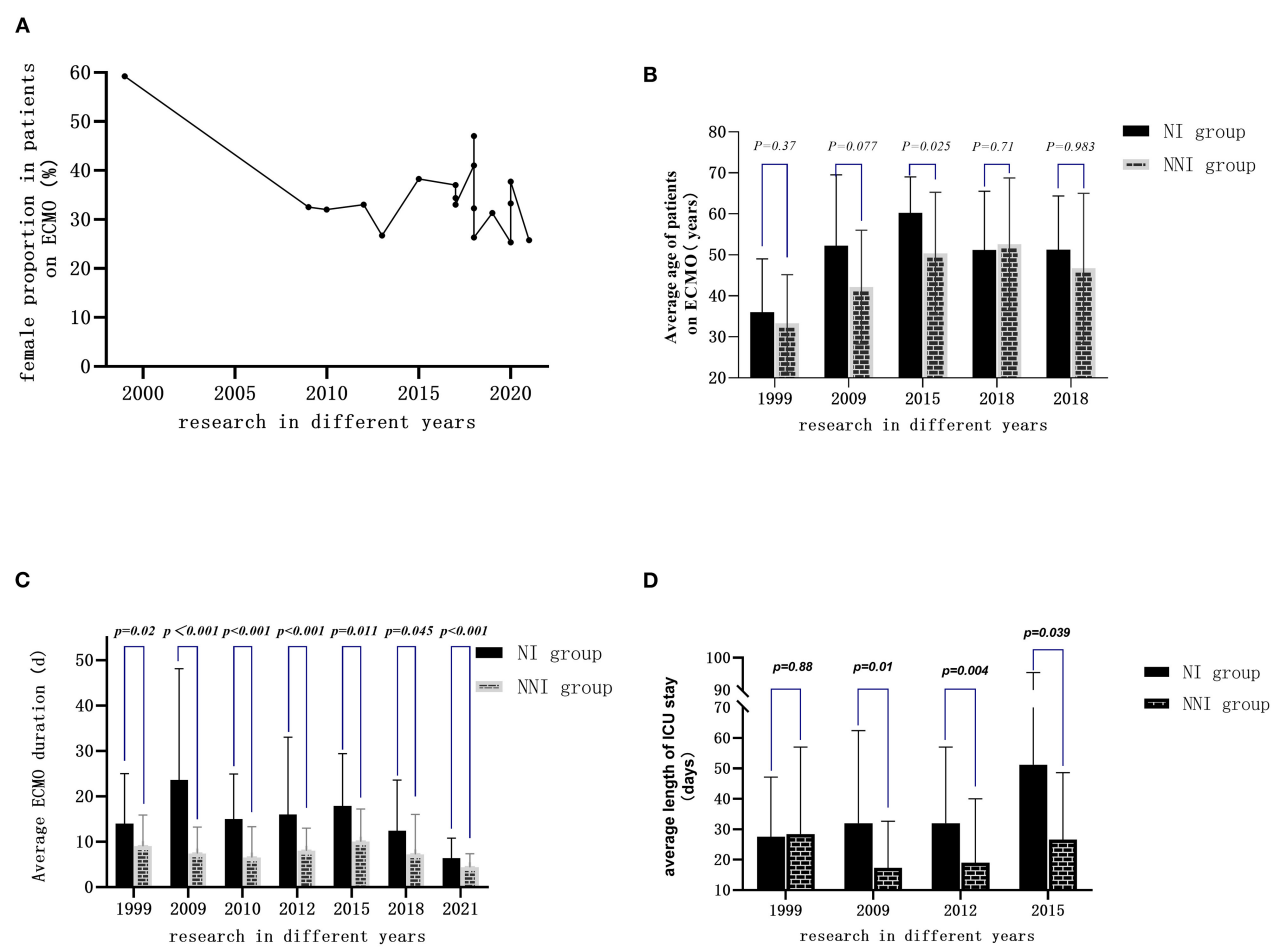
General clinical characteristics of adult patients treated by ECMO in different years: **(A)** Proportion of female patients in most of the studies ranged from 25 to 40%. **(B)** Average age of the patients ranged from 33 to 60 years old. **(C)** Duration of ECMO use extracted from seven studies (expressed as mean ± SD) ranged from 5 days to more than 23 days. **(D)** Length of ICU stay in the infected group was longer than in the non-infected group.

Most of the patients receiving ECMO were male; in most centers, the proportion of female patients was 25–40% (Figure 3A), and the average age was 33–60 years old (Figure 3B). A total of 14 studies reported the duration of ECMO usage (seven of them were expressed as mean ± SD) (Figure 3C). The shortest use time was 5 days, and the longest use time was more than 23 days. The length of ICU stay was 8–32 days. Compared with the non-infected group, the duration of ECMO usage and length of ICU stay in the infected group were longer (Figure 3D).

### Effect of Nosocomial Infection on In-hospital Mortality

Six studies with a quality score of less than 5 were excluded. The remaining 19 studies were included for meta-analysis. A total of 2,676 patients were included in the meta-analysis, including 780 infected patients (433 deaths) and 1,896 non-infected patients (880 deaths).

#### Heterogeneity Test

A heterogeneity test of the 19 studies was performed. The results are as follows: *I*^2^ = 52%, and *P* = 0.005 < 0.1 in the Q test. These results suggest that heterogeneity among the studies included in this study was statistically significant, and that a heterogeneous search was necessary.

#### Sensitivity Analysis to Find Out Causes of the Heterogeneity

A sensitivity analysis was conducted on the 19 studies included in the meta-analysis. The results of this analysis revealed that the study conducted by Kim (2015) (5) had a large impact on the heterogeneity. After removing this study, a heterogeneity test was performed again, and the results revealed that the remaining 18 studies (n = 2,629) had less heterogeneity (*I*^2^ = 27 < 50%, *P* = 0.14>0.1). After excluding the study by Kim et al. (5), a meta-analysis was conducted using the fixed effects model.

#### Meta-Analysis Results of Fixed Effects

**Figure 4:**
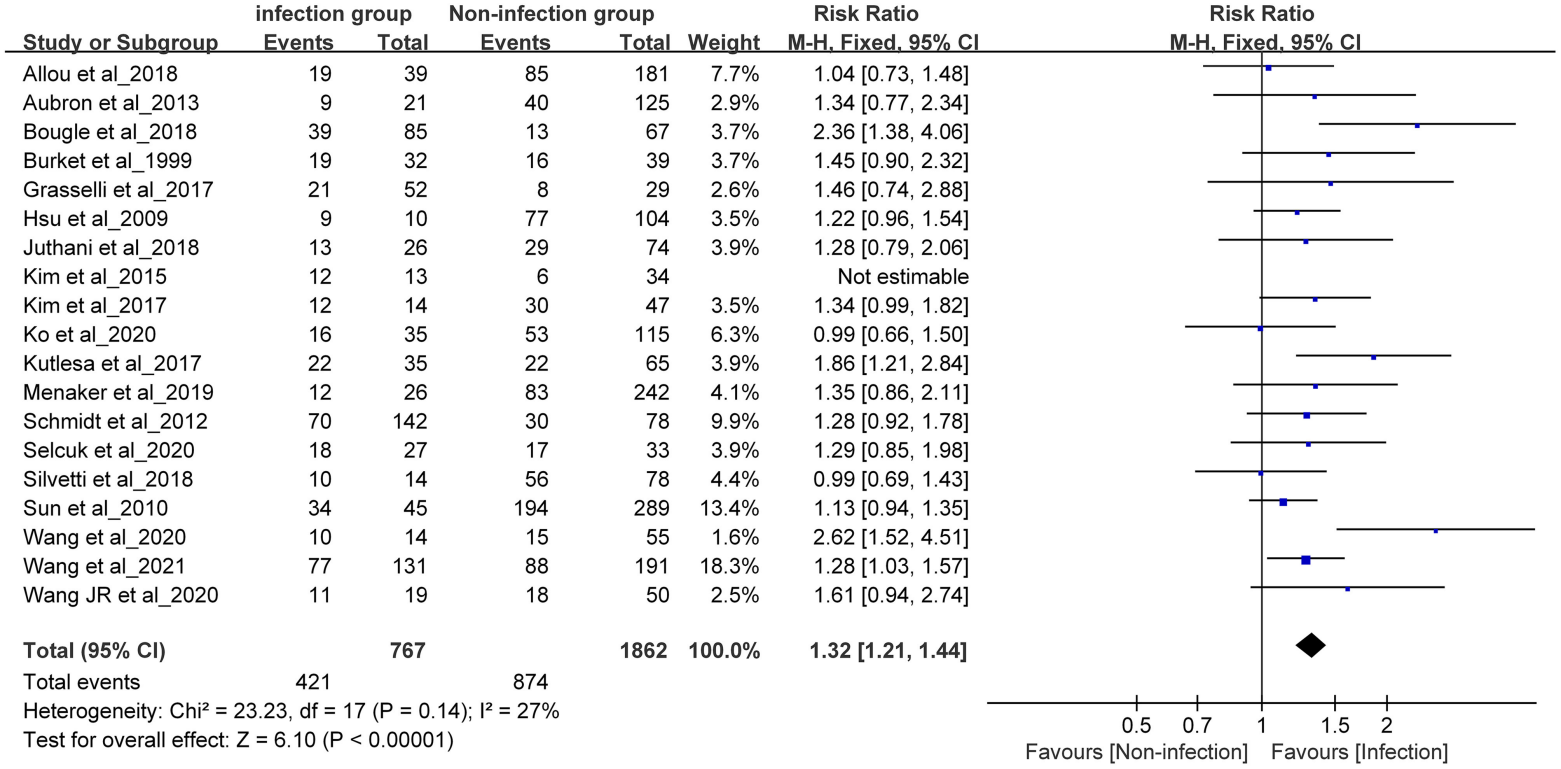
Forest plot was pre-formed with the fixed-effects model after eliminating one study with high heterogeneity.

The summarized RR value of the 18 remaining studies was 1.32, and the 95% CI was 1.21–1.44. This was statistically significant (Z = 6.1, *P* < 0.01) and suggested that the in-hospital mortality in the infection group was significantly higher than that in the non-infection group in adult patients receiving ECMO. The following forest plot (Figure 4) shows the details.

#### Bias Test

**Figure 5:**
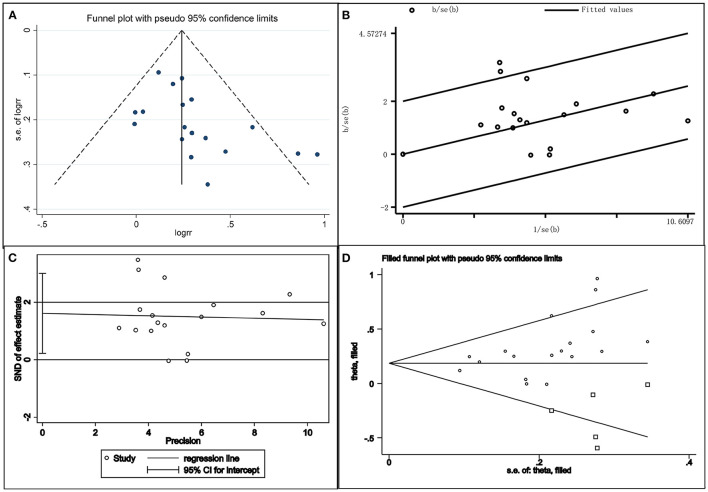
Combination chart of the publication bias test. **(A)** funnel plot; **(B)** radial plot; **(C)** Egger test plot; **(D)** filled funnel plot.

The publication bias of this study was investigated by drawing a funnel plot. The funnel plot was slightly asymmetric; therefore, a star diagram investigation was performed. Although one study affecting the overall heterogeneity was excluded, two studies remained that were beyond the CI. The Egger bias test showed that *t* = 2.46 and *P* = 0.026 < 0.05, indicating that there was a publication bias. The above asymmetric funnel plot was further processed with the shear compensation method that the plot showed five square dots. This suggested that relevant research articles should be included in the future to eliminate publication bias (Figure 5).

### Summary of Risk Factors for Nosocomial Infection in Patients Receiving ECMO

**Figure 6:**
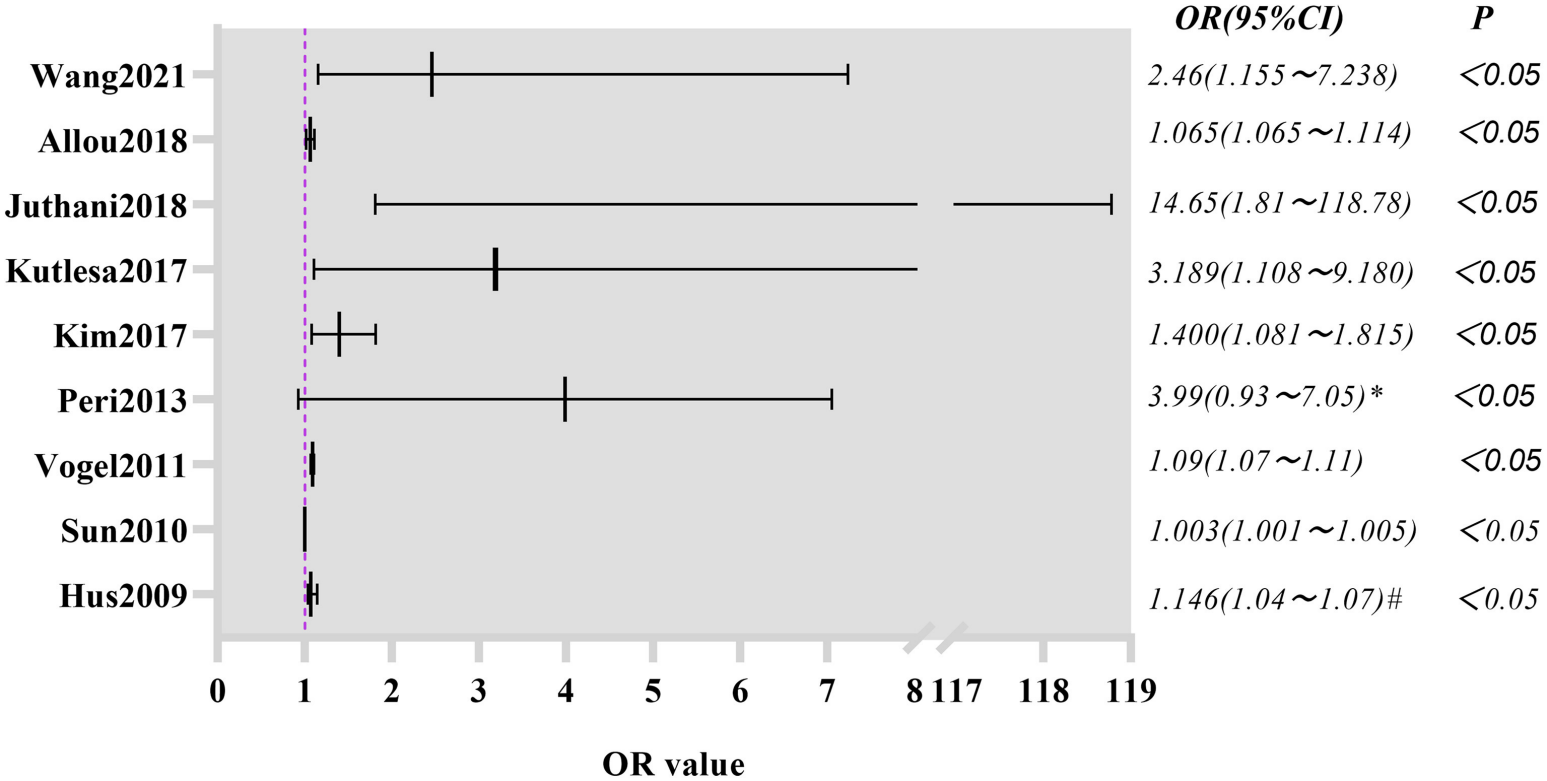
Forest plot of the risk of nosocomial infection due to ECMO duration.

For nosocomial infection, the most reported risk factor in the majority of the studies was the duration of ECMO (4, 7, 10, 12, 13, 15, 17–19) (Figure 6). Second, the Sequential Organ Failure Assessment (SOFA) and the Simplified Acute Physiology Score (SAPS) were considered to be independent risk factors (15, 22, 23). Other risk factors included VV ECMO mode (9, 13), ventilator use time before ECMO removal (9, 14), age of patient (20), and autoimmune disease diagnoses (13). One study concluded that the greater the body mass index, the higher the risk of nosocomial infection (10). However, two other studies reached the opposite conclusion (15, 23).

## Discussion

Nosocomial infection is one of the most common complications in patients receiving ECMO (2). This study focused on nosocomial infection in adult patients receiving ECMO, and clinical epidemiological characteristics of nosocomial infection were summarized and analyzed. The results revealed that there was great heterogeneity in the prevalence, incidence, and in-hospital mortality of nosocomial infection in adult patients receiving ECMO. The results of this study also indicated that respiratory tract infection was the most common nosocomial infection, the pathogenic cause of which was primarily Gram-negative bacteria. There were fewer adult female patients receiving ECMO, and compared with the non-infected group, the duration of ECMO usage and length of ICU stay for patients in the infected group were longer. In addition, the meta-analysis revealed that nosocomial infection increased the risk of death in adult patients receiving ECMO by 32% when compared with non-infected patients. Finally, the results indicated that the risk factors of adult patients receiving ECMO were duration of ECMO usage, disease severity score, age, ventilator use time before ECMO removal, and VV ECMO mode.

There were several reasons for the great heterogeneity of the prevalence of nosocomial infection in adult patients receiving ECMO (29, 30). First, there were differences in the definition of nosocomial infection among different centers, and the main criterion for the early diagnosis of nosocomial infection relied on the results of the pathogenic culture. It is well-known that colonization needs to be excluded if the pathogen culture is positive; however, infection cannot be completely excluded if the pathogen culture is negative. Second, each center lacks a unified antibiotic prevention and treatment strategy and monitoring measures (including routine nursing protocols for prevention of ventilator-associated infection and bloodstream infection). Third, due to the limitation of the sample size, there were not enough well-controlled prospective studies to eliminate the confounding factors, especially for variables with heterogeneous sources, such as etiology or type of operation, severity of the disease, and type of ECMO.

It was reported that bloodstream infection was the most common infection type in neonatal and pediatric patients (31, 32), and the data published online by ELSO showed that the most common pathogen of nosocomial infection was yeast and Staphylococcus aureus; the most common microorganisms isolated from blood culture were Staphylococcus, yeast, and Enterococcus (33). Unexpectedly, lower respiratory tract infection was the most common type of infection in adult patients in this study. Moreover, Gram-negative bacteria were more common pathogens in the adult patients on ECMO with nosocomial infection. Although the situation varies from center to center, the etiological distribution of nosocomial infection during ECMO is similar to that without ECMO support (29).

The prevalence of nosocomial infection in patients with ECMO treatment less than 1 week, during 8 to 14 days, more than 2 weeks was 6.1%, 15.7%, 30.3%, respectively (3). It remains controversial whether nosocomial infection leads to longer ECMO support or whether longer ECMO support leads to an increased risk of nosocomial infection. These two factors were reciprocal causation. The extension of ECMO support increased the chance of nosocomial infection; in turn, nosocomial infection resulted in longer ECMO support time (33).

Older age, severity score of illness and underlying autoimmune disorders were regarded as risk factors for nosocomial infection during ECMO support. However, Grasselli et al. claimed that younger age, not elder age, was an independent risk factor of nosocomial infection in patients on ECMO treatment (6). The evidence was still low because of some limitations from these retrospective single-center studies. Much more systematic investigations should be performed in the future (33).

Early studies, including 5,000 cases based on the ELSO database, showed that neonates on ECMO treatment with nosocomial infection had significantly higher mortality (34, 35). As mentioned above, there was no consistent association between nosocomial infection and mortality in adult patients receiving ECMO treatment. As expected, in this study, the meta-analysis revealed that nosocomial infection increased the risk of in-hospital mortality (by 32%) in adult patients receiving ECMO. This is consistent with the results of a large sample study published by ELSO in 2011 (pediatric patients were included) (4). It is speculated that the included studies were single-center retrospective ones and that most of the included studies did not show the adverse effect of nosocomial infection on mortality because of the small sample size.

This study has several limitations. First, the studies included spanned a 22-year time frame, and great changes have taken place in equipment, consumables, and medical technology during that time span. This resulted in poor vertical comparability. Therefore, only a narrative review is made, and there is no meta-analysis of prevalence and incidence. Second, all the included studies were retrospective. Some of the studies did not report the length of ICU stay and length of the hospital stay, and the heterogeneity is high. Third, the long collection cycle of the ECMO treatment cases, small sample size, and low statistical efficiency resulted in inherent defects in the methodology. Fourth, in this study, there were 23 studies with patients aged ≥ 18 years old, but in one study, the patients included were ≥ 16 years old, and in another study, the patients included were ≥ 17 years old. Therefore, the age of inclusion in this study did not follow the routine rule; that is, the age of patients should be ≥ 18 years old. However, this did not affect the outcome of this study.

## Conclusion

In general, adult patients treated by ECMO had high prevalence and incidence of nosocomial infection. The prevalence and incidence of nosocomial infection varied from center to center. For the first time, the meta-analysis in this study made it clear that nosocomial infection increased the in-hospital mortality by 32% in adult patients receiving ECMO treatment. Compared with patients without nosocomial infection, the ECMO use time was longer when patients combined with nosocomial infection, lower respiratory tract infection was the most common nosocomial infection, and the pathogenic cause was primarily gram-negative bacteria. The prevention and treatment of nosocomial infection during ECMO support is an important clinical topic that cannot be ignored. Improvement in diagnostic measures, monitoring, target treatments, and preventive measures must be implemented.

## Author Contributions

XH and XL: conception and design of the research. XL, LW, and HW: analysis and interpretation of the data and statistical analysis. XL: obtaining financing and writing of the manuscript. XL and LW: acquisition of data. XH and HW: critical revision of the manuscript for intellectual content. All authors read and approved the final draft.

## Funding

This study was funded by the Sailing Program of Key Medical Specialty of Beijing Hospitals Authority in 2021: for critical care medicine (fund number for extracorporeal life support: ZYLX202111). The funding body had no role in the design of the study, collection, analysis, interpretation of the data, and in writing the manuscript.

## Conflict of Interest

The authors declare that the research was conducted in the absence of any commercial or financial relationships that could be construed as a potential conflict of interest.

## Publisher's Note

All claims expressed in this article are solely those of the authors and do not necessarily represent those of their affiliated organizations, or those of the publisher, the editors and the reviewers. Any product that may be evaluated in this article, or claim that may be made by its manufacturer, is not guaranteed or endorsed by the publisher.
